# Stratigraphy of the Middle-Upper Permian and Lowermost Triassic at Chaotian, Sichuan, China: Record of Late Permian double mass extinction event

**Published:** 2004-01-01

**Authors:** Yukio Isozaki, Jianxin Yao, Tetsuo Matsuda, Harutaka Sakai, Zhansheng Ji, Noriei Shimizu, Noritada Kobayashi, Hodaka Kawahata, Hiroshi Nishi, Masao Takano, Tomomi Kubo

**Affiliations:** *1) Dept. Earth Science and Astronomy, University of Tokyo, 3-8-1, Komaba, Meguro-ku, Tokyo 153-8902, Japan; *2) Chinese Academy of Geological Science, Baiwanzhuang, Beijing 100037, China; *3) Kyoei Consult Co., Oh-Izumi, Toyama 939-8093, Japan (deceased on April 23, 2002); *4) Dept. Earth Environ. Sci., Kyushu Univ., Ropponmatsu, Chuo-ku, Fukuoka 810-0044, Japan; *5) Dept. Marine Geol., Geological Survey of Japan, Higashi, Tsukuba, Ibaraki 305-8567, Japan; *6) Dept. Environ. Sci., Nagoya Univ., Furoucho, Chikusa-ku, Nagoya, Aichi 464-8602, Japan

**Keywords:** Mass extinction, Permo-Triassic boundary (P-T), Guadalupian-Lopingian (G-L) boundary, acidic volcanism, South China

## Abstract

Precise stratigraphic analysis of Middle-Upper Permian and Lower Triassic sequence at Chaotian in northern Sichuan, China, identified two remarkable mass extinction horizons, one at the Middle-Upper Permian (Guadalupian-Lopingian; G-L) boundary and the other at the Upper Permian-Lower Triassic (P-T) boundary. Across each of the boundaries, biodiversity declined sharply in fusulinid, rugose coral, brachiopod, ammonite, conodont, and radiolaria. Both boundaries are characterized by two biohorizons, i.e., one marked by major extinction of pre-existing fauna and the other by the first appearance of younger fauna. It is noteworthy that a peculiar rhyo-dacitic tuff bed occurs at each of the extinction horizons. Thus the Late Permian biosphere was strongly affected twice by highly explosive, severe volcanism. Regional correlation of the G-L and P-T boundary tuff beds throughout South China, and partly to Japan, positively suggests a cause-effect link between large-scale explosive volcanism and mass extinction.

## Introduction

The Upper Permian sedimentary rocks in South China called the Lopingian have recently drawn special attention because it provides the best stratigraphic record for the interval immediately before the P-T boundary mass extinction. Special emphasis has been given to the Lopingian since the geological significance of another major mass extinction event at the base of the Lopingian, i.e., the Guadalupian-Lopingian (G-L) or Middle-Upper Permian boundary, was pointed out.1),2) Fossil records in South China indicate that a rapid faunal turnover across the G-L boundary was as significant as that across the P-T boundary. Thus the Lopingian represents a unique time interval punctuated on both sides by major extinction events.

**Fig. 1:**
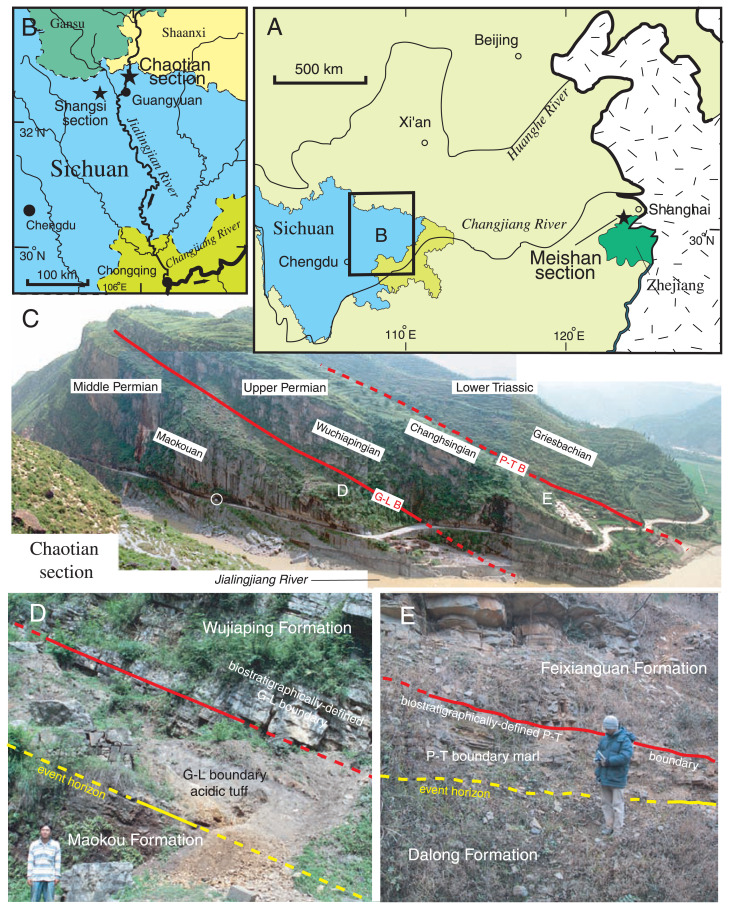
The Chaotian section in northern Sichuan, China. A, B: Locality index of the Chaotian section, C: The Chaotian section viewed from the western side of the Jialingjiang River. A car on the road (in a circle) for scale, D: The outcrop of the Guadalupian-Lopingian (G-L) boundary between the Maokou and Wujiaping Formations. Note the 2m thick boundary acidic tuff bed, E: The outcrop of the Permian-Triassic (P-T) boundary between the Dalong and Feixianguan Formations. Note the 1.4 m thick boundary marl bed.

For deciphering the global environmental change relevant to the double mass extinction events of the Late Permian, a detailed stratigraphic analysis is inevitable in a continuous, fossiliferous Lopingian section with a simple tectono-sedimentary history. A favorable shelf carbonate sequence with abundant fossils is exposed at Chaotian in northern Sichuan, South China (Fig. 1A, B), in which the G-L and P-T boundaries are clearly exposed. This article reports a short summary of our stratigraphical research at Chaotian since 1998 with special emphasis on the G-L and P-T boundaries.

## Geologic setting

Northern Sichuan and southern Shaanxi at the northwestern part of South China (Yangtze) craton accommodate an extraordinarily thick pile of the Paleozoic and Mesozoic sedimentary rocks.3) The Permian rocks of the region consist mainly of shallow marine carbonates with subordinate amounts of mudstone. This region was totally covered by platform carbonates before the end of the Guadalupian,4) while in the Lopingian, the carbonate platform became localized, and partly changed into slope to deeper basins of mudstone facies.

The study section at Chaotian in northern Sichuan crops out along the Jialingjiang River, in a narrow gorge called Mingyuexia, ca. 20 km to the north of Guangyuan city (Fig. 1B, C). The Chaotian section, over 200 m thick, consists mainly of fossiliferous, shallow marine carbonates of slope to basin facies, spanning from the Guadalupian, via Lopingian, to the Griesbachian (the earliest Triassic). Abundance in bioclasts of shallow-water organisms suggests proximity to carbonate platform, while frequent intercalation of chert lens/nodule indicates a slightly deeper environment than platform. We studied these Middle Permian to earliest Triassic rocks exposed along the eastern bank of the river, on the southern limb of an anticline.

## Stratigraphy

**Fig. 2:**
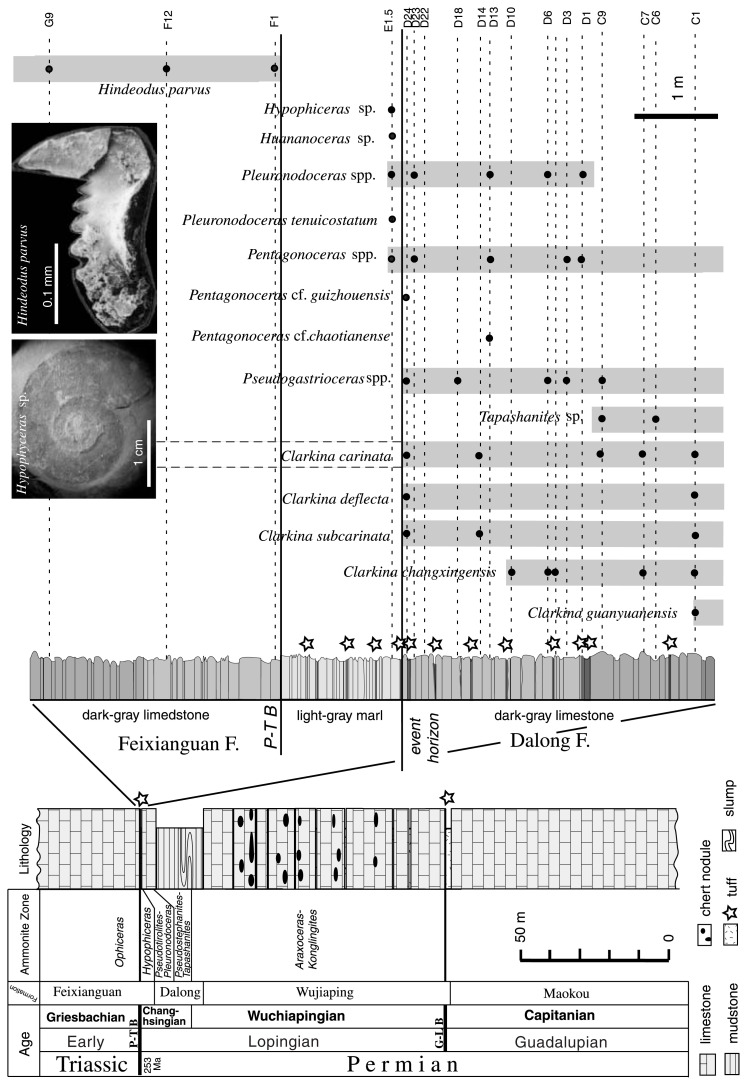
Stratigraphy of the Middle-Upper Permian to Lower Triassic sequence of the Chaotian section. Left: Stratigraphic column of the whole section, Right: Detailed columnar section across the P-T boundary horizon with photomicrographs of the biostratigraphically diagnostic taxa. Note the G-L and P-T boundary horizons are characterized by occurrence of acidic tuff beds.

The Chaotian section consists of the Maokou Formation (Guadalupian), Wujiaping Formation (Wuchiapingian), Dalong Formation (Wuchiapingian to Changhsingian), and Feixianguan Formation (Griesbachian) in ascending order (Fig. 2). The G-L and P-T boundaries are recognized at the Maokou/Wujiaping contact and at the Dalong/Feixianguan contact, respectively (Fig. 1C–E).

The **Maokou Formation**, over 75 m thick, is composed mainly of thickly bedded, dark-gray, bituminous, bioclastic limestone. Wackestone and lime mudstone predominates, containing various Tethyan fossils including fusulinid, brachiopod, gastropod, rugose coral, calcareous alga, and crinoid. The uppermost part of the formation consists of a 10 m thick gray wackestone and overlying 10 m thick, black, calcareous mudstone.

The following fossils are newly obtained from this formation.

Fusulinid: *Yabeina* sp., *Verbeekina* sp., *Pseudodoliolina pseudolepida*, *Chusenella* sp., Conodont: *Jinogondolella postserrata*, *J*. *wilcoxi*, *Hindeodus minutus*

On the basis of the conodonts, the age of the formation is regarded as the Capitanian (Late Guadalupian).

The **Wujiaping Formation**, 68 m thick, is composed mainly of dark-gray limestone, mostly of wackestone and packstone. It contains various fossils including fusulinid, smaller foraminifer, calcareous algae, brachiopod, gastropod, crinoid, rugose coral, and conodont. Ammonites occur rarely.

The lowermost part of the formation is composed of a unique non-calcareous unit composed of acidic tuff (2 m thick) and tuffaceous sandstone (1 m thick) that is overlain by thinly bedded bioclastic limestone (Fig. 1D). The main part (49 m thick) consists of dark-gray massive limestone with abundant chert nodule/lens. The upper part consists of brachiopod-enriched gray limestone (2 m thick), fine-grained, argillaceous black limestone (3 m thick), and nodular, micritic limestone with chert nodule (6 m thick), and argillaceous black limestone (2 m thick), in ascending order.

Representative fossils from this formation are listed below (* 5); ** 6); *** this study).

Conodont: Clarkina orientalis**, C. liangshanensis**, C. guanyuanensis***, Fusulinid*: *Codonofusiella schuberteroides*, *C. asiatica*, *Reichelina* aff. *pulchra*, *Palaeofusulina simplex*, Rugose coral*: *Liangshanophyllum wengchengense*, *Waagenophyllum simplex*, Ammonite*: Araxoceratidae gen. et sp. indet.

These fossils consistently indicate the Wuchiapingian (Early Lopingian) age for this formation.

The **Dalong Formation**, 26 m thick, consists of bituminous, black mudstone (22 m thick) and overlying micritic, gray limestone (4 m thick). The black mudstone is bedded, and it intercalates thin (10–20 cm) layers/lenses of marl. Slump beds occur at two horizons within this mudstone. The uppermost (2 m thick) part of the formation comprises gray, micritic limestone rhythmically bedded in 5 cm with nodular surface. The mudstone and limestone are enriched in ammonite, conodont, and radiolaria. Thin layers of acidic tuff, less than 10 cm thick, occur at 8 horizons in the uppermost part (fig. 2).

Representative fossils from this formation are listed below (* 5), ** 6); *** this study) with indication of the ages.

1. lower mudstone (Araxoceras-Konglingites Zone of the late Wuchiapingian)
  Ammonite: *Konglingites* sp.*(age indicator), *Jinjiangoceras* sp.*, Conodont: Clarkina orientalis**
2. upper mudstone (Pseudostephanites-Tapashanites Zone of the early Changhsingian)
  Ammonite (including the zone indicators): Tapashanites floriformis*, T. chaotianensis*, T. costatus**, Sinoceltites curvatus*, Changhsingoceras sichuanese*, *Pseudostephanites?* sp.**, Conodont: Clarkina subcarinata***, C. changxingensis***, C. deflecta***, C. postwangi***, Brachiopod: Dictyoclostus gratiosus*, *Waagenites* cf. soochowensis*, *Leptodus* sp.**
3. uppermost limestone (2 m thick)/(Pseudotirolites-Pleuronodoceras Zone and Rotodiscoceras Zone of the late Changhsingian)
  Ammonite: Pleuronodoceras mapingensis*, Pseudotirolites asiaticus*, Chaotianoceras modestum*, *Pentagonocers* cf. guizhouensis***, *Rotodiscoceras* sp.*, *Pseudogastrioceras* sp.*, Conodont: Clarkina changxingensis**, C. deflecta**, C. subcarinata***, C. meishanensis zhangi***

The **Feixianguan Formation**, over 30 m thick, consists mainly of thinly bedded, light-gray, micritic limestone. The lowermost 1.4 m is made up of a unique bed of olive-gray, faintly laminated marl that is unique to this horizon in the Chaotian section. This marl is almost barren of fossil, except for the basal 5 cm interval that yields small ammonites and bivalves. The marl is composed mainly of clay minerals (mostly illite) without calcareous/phosphatic bioclast or radiolarian test observable in thin section. Thin layers of acidic tuff, less than 3 cm thick, occur at 4 horizons (Fig. 2). The overlying limestone is thinly bedded, partly interbedded with thin marl. Fossils from this formation are listed (** 6); *** this study) and their ages are briefly mentioned below.

1. marl (uppermost Changhsingian)
  Ammonite (all smaller than 3 cm in diameter): Pleuronodoceras tenuicostatum***, *Pentagonoceras* sp.***, *Huananoceras* sp.***, *Hypophiceras* sp.***, Bivalve: Claraia wangi**
  The newly found ammonites from the lowermost marl represent Changhsingian holdover taxa that characterize the Hypophiceras Zone of the latest Changhsingian in South China. Further check is needed for the precise horizon of previously reported Triassic ammonite *Ophiceras* sp. from the marl.6)
2. bedded limestone (earliest Griesbachian)
  Conodont: Hindeodus parvus***, Brachiopod: *Lingula* sp.***

The occurrence of *H. parvus* without accompanying *Isarcicella isarcica* indicates that the lowermost limestone of the Feixianguan F. belongs to the Hindeodus parvus Zone of the earliest Griesbachian age.

## Discussion

On the basis of the litho- and biostratigraphy of the Chaotian section, we discuss geological implications of the two extinction-relevant boundaries.

### G-L boundary

The fossil data indicate that the G-L boundary at Chaotian is placed between the Maokou Formation and Wujiaping F. (Fig. 2). At the formation boundary, a unique clayey tuff bed, 2 m thick, occurs as a boundary marker (Fig. 1D). This boundary tuff bed rests conformably on the Maokou F. but appears to have truncated the continuous deposition of the latter. The large-shelled Guadalupian fusulinids, rugose corals, brachiopods and conodonts declined abruptly in the uppermost Maokou F. Thus the top of the Maokou F. corresponds to the event boundary horizon marked by a major extinction. On the other hand, the biostratigraphically-defined G-L boundary is placed at the top of the boundary tuff, where the Lopingian fauna first appeared. The *Codonofusiella-Reichelina* (fusulinid) assemblage predominates in the lowermost Wujiaping F. that consists of gray bioclastic limestone with abundant calcareous algae.

The boundary clayey bed is gray when fresh but is often weathered into yellow to reddish-gray, soft bentonite. Analyses by XRD, XRF and SEM clarified that the clay bed was originated from a volcanic ash of rhyolitic to dacitic composition. It is composed mainly of mixture of illite-montmorillonite probably derived from volcanic glasses, with abundant euhedral phenocrysts of apatite, zircon, plagioclase, and bipyramidal (high-temperature type) quartz. In general, euhedral zircon and bipyramidal quartz rarely occur in basalt.

The occurrence of a unique tuff bed at the G-L boundary positively suggests an intimate causal link between the mass extinction and volcanism in the following aspects. First, the abrupt termination of the fossiliferous Maokou limestone suggests that sudden ash fall may have destroyed the favorable ecological condition for the Guadalupian shallow marine organisms. Second, the 2 m thick G-L boundary tuff is significant in utility in tephrachronologic correlation and also in estimation on magnitude of volcanism. The thickness over 2 m after compaction is exceptionally large because it is two orders of magnitude greater than other tuff beds recognized not only in the Chaotian section but also in the whole Permian to Triassic sequences in South China. This large thickness may imply either proximity to source or unusually intense volcanism. Third, the rhyodacitic composition of the tuff, with euhedral phenocrysts of zircon, plagioclase, and high temperature-type quartz in particular, indicates its origin from highly explosive, felsic volcanism.

According to our reconnaissance study, the G-L boundary tuff bed at Chaotian is well correlated with the Wangpo bed at the base of the Wujiaping F. in the stratotype section in Lianshan, Shaanxi.7) In addition, we have recognized the identical tuff bed at the same horizon in several other sections in South China; e.g. the Shangsi,3) Huaying, and the Emeishan sections in Sichuan plus the Penglaitan section in Guangxi. Thus a thick rhyo-dacitic ash bed must have covered the entire South China at the G-L boundary timing.

The acidic tuff across the G-L boundary has been recently found also in paleoatoll limestone and deep-sea chert in Japan.8) This means that the G-L boundary acidic volcanism influenced not only South China but also a mid-oceanic realm of the eastern superocean Panthalassa. Moreover, the onset of the deep-sea anoxia (superanoxia) in the superocean apparently synchronized with the G-L boundary time.9)

Consequently, these lines of evidence all indicate that a reorganization of global environment has started at the G-L boundary, probably triggered by acidic volcanism of unusually large scale. The site of responsible volcanism, however, is still unknown. The apparently coeval Emeishan basalt in Sichuan and Yunnan representing a continental flood basalt suite is often nominated as a cause of the G-L boundary crisis10),11) but its basaltic nature cannot be correlated directly with the acidic geochemistry of the G-L boundary tuff.

### P-T boundary

The fossil data indicate that the P-T boundary of the Chaotian section is placed in the basal Feixianguan Formation (Fig. 2). A unique marl unit (1.4 m thick) at the basal Feixianguan F. serves as the boundary marker (Fig. 1E). The top of the Dalong limestone represents the event boundary horizon marked by the extinction of many Changhsingian taxa. The sharp lithologic change from the Dalong limestone to the Feixianguan marl coupled with the extinction marks an event that involved rapid collapse in marine productivity across the P-T boundary. The boundary marl is almost barren of fossil, except the lowest part yielding tiny (less than 3 cm in diameter) ammonites that represent the Changhsingian holdover taxa.

The boundary marl represents an interval of strong environmental stress. The total extinction of the Permian radiolarians suggests appearance of a harsh environment because these Paleozoic zooplanktons have played major role in marine primary productivity in the superocean Panthalassa and Tethys. In addition, the sudden size reduction in ammonite and brachiopod plus the occurrence of a typical disaster taxon *Lingula*12) from the lowermost Feixianguan F. further indicates the environmental deterioration. These observations consistently suggest that a strong environmental stress, particularly for those secreting calcareous and siliceous shells, appeared suddenly at the final moment of the Changhsingian.

The bedded limestone above the boundary marl yields the index Griesbachian conodont *Hindeodus parvus* (Fig. 2). The biostratigraphically-defined P-T boundary is set at the top of the marl because the FAD (First Appearance Datum) of *H. parvus* is widely accepted as the prime criterion for the P-T boundary.13)

It is worth noting that the P-T boundary at Chaotian is characterized by frequent intercalation of rhyo-dacitic tuff beds (Fig. 2). In the study section, we identified 12 tuff beds from 7 m interval around the P-T boundary, and these thin beds of tuff are concentrated particularly in the uppermost Dalong F. (8 beds) and lowermost Feixianguan F. (4 beds). Between the top-most Dalong limestone and the overlying marl, for example, a 5 cm thick prominent acidic tuff bed occurs. Analyses by XRD, XRF and SEM revealed that these acidic tuff beds have almost identical mineralogical and geochemical characteristics to those of the G-L boundary tuff; rhyo-dacitic in composition and bearing euhedral phenocrysts of quartz, apatite, and zircon.

Similar mode of occurrence of tuff beds across the P-T boundary is confirmed also at Shangsi, Meishan, and in many other sections in South China. In the Shangsi and Meishan sections in particular, almost identical tuff beds were recognized in the same interval across the P-T boundary3),14) and the ones immediately below and above the FAD of *H. parvus* were chronometrically dated to be 251–253 Ma.15),16) Thus it seems likely that rhyo-dacitic ash layers may have covered the entire South China across the P-T boundary.

Judging from the rhyo-dacitic geochemistry, fine-grained nature, and extensive distribution, the P-T boundary volcanic ash beds were probably originated from highly eruptive volcanoes somewhere, although possible source of such rhyo-dacitic volcanism at the P-T boundary is still unknown. Again, the apparent age coincidence is emphasized for eruption of the Siberian traps in Russia,17)–19) however, the geochemistry, is apparently inconsistent with that of the P-T boundary tuff beds, just like the similar case for the G-L boundary.

## Conclusion

At the Chaotian section in northern Sichuan, detailed stratigraphic analysis of the Middle-Upper Permian and Lower Triassic sequence revealed two remarkable mass extinction horizons, one across the G-L boundary and the other across the P-T boundary. It is noteworthy that at these horizons there are records of intense rhyo-dacitic volcanism. This indicates an intimate cause-effect link between the volcanism and environmental change relevant to the Late Permian double mass extinction event.
